# Effects of achievement goals on learning interests and mathematics performances for kindergarteners

**DOI:** 10.3389/fpsyg.2023.1156098

**Published:** 2023-05-17

**Authors:** Chung Chin Wu

**Affiliations:** Early Childhood Education, National Pingtung University, Pingtung, Taiwan

**Keywords:** achievement goal, goal orientation, kindergartener, learning interest, mathematics performance

## Abstract

**Background:**

Studies have investigated the effects of achievement goals on learning interests and mathematics performance above the elementary-school level. However, few studies have explored this topic among kindergarteners based on sound theoretical frameworks.

**Methods:**

Through the enrollment of 15 kindergarten teachers and 180 kindergarteners, this study re-validated newly developed measurements of kindergarteners’ achievement goals and learning interests and used these measures to further clarify the effects of achievement goals on learning interests and mathematics performances using structural equation modeling.

**Results:**

The results indicate that (1) task-approach goals have positive effects on situational interest and advanced arithmetic performance, whereas task-avoidance goals have positive effects on individual interest. (2) Self-based goals have null effects on most learning interests and mathematics performance, but they have significant negative effects on numbering and counting performance. However, most of these null effects represent negative tendencies. (3) Other-approach goals have positive effects on situational interest and basic arithmetic performance, whereas other-avoidance goals have null effects on these outcomes but have an almost significant positive effect on numbering and counting performance. (4) Task-based goals and self-approach goals are generally beneficial for learning interests and mathematics performance.

**Conclusion:**

These results suggest that task-based goals and other-approach goals may be implemented with consideration of the potential long-term detrimental effects of social comparison on learning outcomes. Furthermore, possible negative effects of self-based goals must be monitored to prevent them from undermining learning outcomes. This study revealed consistent, inconsistent, and new evidence that, respectively, verifies, complements, and contradicts findings on the learning outcomes of students above the elementary-school level.

## Introduction

In practice, teachers are interested in cultivating students’ motivation toward adaptive achievement to benefit learning outcomes (e.g., learning interest and mathematics performance). In recent years, researchers have attempted to identify which types of achievement motivation can contribute to these results. Achievement goal theory has been proposed to conceptualize achievement motivation and has been widely discussed in achievement motivation literature over the past three decades. Several theoretical viewpoints regarding achievement goals have been proposed and adopted for different research contexts (Huang, 2012).

A majority of existing studies have been conducted with dichotomous (i.e., mastery-approach goal and performance-approach goal), trichotomous (i.e., mastery-approach goal, performance-approach goal, and performance-avoidance goal), or 2 × 2 achievement goal theoretical frameworks (i.e., mastery-approach goal, mastery-avoidance goal, performance-approach goal, and performance-avoidance goal, which orients individuals to pursue achievement, to avoid failing to achieve the task-requirement, to aim at outperforming others, and to avoid performing inferior to others, respectively). Several inconsistent effects of identical or similar achievement goals on learning outcomes have been found. For example, some researchers have found that the mastery-approach goal-oriented students to master the learning content, which enhanced their performance in mathematics; others have found null or negative effects on mathematics performance (Wolters, 2004; Anderman and Patrick, 2012; Plante et al., 2013; Seaton et al., 2014). In addition, most of these studies were conducted above the elementary-school level. Few studies have investigated the effects of achievement goals on learning outcomes for young children below the elementary-school level, and existing studies have usually retrieved one or two achievement goals from the above theoretical frameworks for variables or predictors. For example, Zee et al. (2021) investigated the longitudinal effects of task motivation on mathematics performance, but the effects of achievement goals on learning outcomes for young children were unclear. Consequently, the advantages and disadvantages of holding certain achievement goals remain ambiguous, especially for young children (e.g., kindergarteners). In addition, the inconsistencies and uncertainties of former findings may be increased due to the lack of a consensus concerning the definition of mastery-based goals (i.e., mastery goal and mastery-avoidance goal).

Recently, Elliot et al. (2011) argued that two different goal foci (i.e., absolute task requirement and intrapersonal relative standards) are confounded together to define mastery-based goals in the 2 × 2 achievement goal theoretical framework. They proposed a new 3 × 2 achievement goal model comprised of six achievement goals by differentiating mastery-based goals into task-based goals (i.e., task-approach goal and task-avoidance goal, their meanings of which are identical to mastery-approach goal and mastery-avoidance goal, respectively) and self-based goals (i.e., self-approach goal and self-avoidance goal, which orient the individuals to pursue better performance and to avoid not performing worse than they have done in the past, respectively); performance-based goals from the 2 × 2 achievement goal framework were still included in this new model but were replaced with similar names (i.e., other-approach goal and other-avoidance goal, the meanings of which are identical to performance-approach and performance-avoidance goal, respectively). Many studies have found evidence supporting this new model, where task- and self-based goals have been found to have different effects on learning outcomes (e.g., Diseth, 2015; Danthony et al., 2020). However, the effects of these achievement goals on learning outcomes are inconclusive in school-age samples, and these effects are especially unclear in samples below the age of six.

Classroom structures that teachers emphasize in their class (e.g., teacher values mastery of learning tasks) may cultivate corresponding achievement goals for students, which, in turn, can affect their learning outcomes (Wolters, 2004). However, classroom structures in kindergarten are less clear compared with elementary school. Findings on the effects of achievement goals on learning outcomes are also unclear. Thus, more studies on kindergarteners are needed to ensure robust conclusions can be made about the effects of achievement goals on learning outcomes. Corresponding findings may be beneficial for kindergarten teachers in terms of identifying which teaching strategies promote adaptive goals (and which eliminate maladaptive goals) for kindergarteners. Among these learning outcomes, learning interest is critical for kindergarteners, and mathematics performance in early years has been found to have profound effects on later academic success (Jordan et al., 2010; Fisher et al., 2012). However, these two areas have been insufficiently studied using conventional frameworks and the new achievement goal framework. With this in mind, the current study aims to advance the understanding of the effects of the newly proposed six-factor achievement goals on learning interest and mathematics performance.

### Advances of achievement goal theory

Achievement goal theory suggests that achievement motivation and achievement-related outcomes can be understood by clarifying the reasons for students engaging in learning activities (Wolters, 2004). Initially, two types of achievement goals were identified: mastery goal and performance goal. The mastery goal motivates students to develop their competence by means of task proficiency, whereas the performance goal orients students to demonstrate their competence by outperforming others (Pintrich, 2000). Elliot and Church (1997) argued that, from the dichotomous theoretical viewpoint, the performance goal may be grounded in different achievement motivations (i.e., success approach or failure avoidance), and thus it should be differentiated into the performance-approach goal and performance-avoidance goal. Empirically, the performance-approach goal and performance-avoidance goal have been demonstrated to predict different learning outcomes. Consequently, a trichotomous framework of achievement goal theory was developed (i.e., mastery-approach goal, performance-approach goal, and performance-avoidance goal). Later, the mastery-approach goal in the trichotomous framework was further subdivided in terms of achievement motivation. A 2 × 2 achievement goal framework was proposed according to how competence is defined and valanced. In this theoretical framework, mastery and performance goal foci are, respectively, defined based on an absolute/intrapersonal standard (e.g., task requirement/what they have done in the past) and an interpersonal standard (e.g., others’ performance), and competence is positively and negatively valanced, respectively, based on approaching success and avoiding failure. Accordingly, the four-factor achievement model consists of a mastery-approach goal, a mastery-avoidance goal, a performance-approach goal, and a performance-avoidance goal (Elliot and McGregor, 2001). Huang (2012) conducted a meta-analysis of existing dichotomous, trichotomous, and 2 × 2 achievement goal frameworks, concluding that the 2 × 2 achievement goal framework was the best theoretical viewpoint for understanding students’ academic achievement above the elementary-school level.

Elliot et al. (2011) argued that either an absolute standard or intrapersonal standard could be used to define mastery-based goals, but these two standards may vary from one another, and should thus be differentiated into task- and self-based goals. Elliot et al. proposed a 3 × 2 achievement goal model based on three different standards (i.e., absolute, intrapersonal, and interpersonal) and two achievement motivations to define and valance competence. They demonstrated that, compared with alternative models (e.g., dichotomous, trichotomous, and 2 × 2 achievement goal models), the 3 × 2 achievement goal model was the best theoretical model to understand college students’ achievement motivation. This model consists of six factors: task-approach goal, self-approach goal, other-approach goal, task-avoidance goal, self-avoidance goal, and other-avoidance goal. The task-approach goal and task-avoidance goal orient students to pursue success through task mastery and to avoid not achieving the task requirement, respectively. The self-approach goal and the self-avoidance goal motivate students to pursue performing better than and to avoid not performing worse than what they have done in the past, respectively. The other-approach goal and the other-avoidance goal urge students to outperform and avoid not performing worse than others, respectively (Hunsu et al., 2021).

Many studies have favored the 3 × 2 achievement goal model across countries and subject domains (Wu, 2014; Gillet et al., 2015; Hoi, 2016; Mascret et al., 2017; Méndez-Giménez et al., 2017, 2018; Wang et al., 2017; Hidayat et al., 2018; Danthony et al., 2020; Hunsu et al., 2021). However, this model has mainly been applied to school-aged samples, the exception being Wu (2022b), who developed an instrument to measure kindergarteners’ achievement goals based on the 3 × 2 achievement goal framework, and this model as preliminarily demonstrated to be the best theoretical model for understanding kindergarteners’ achievement goals. However, the results are far from conclusive. More studies are needed to re-examine the 3 × 2 achievement goal model and its effects on learning outcomes for kindergarteners.

### Learning interest and its empirical status

Learning interest contains both affective and cognitive components (i.e., positive affections, cognition, and concentration level) when students are engaged in learning activities. The affective and cognitive components of learning interest can differ in terms of degree or proportion. Initially, the affective component exerts a greater impact than the cognitive component; however, as learning interest develops, the influence of the cognitive component increases (Hidi et al., 2004). Moreover, in the early stages, learning interest incorporates two dimensions: situational interest and individual interest. Situational interest is triggered by (and varies according to) learning context or instructional design (e.g., interesting course contents); it can transform into individual interest once it is maintained for a period of time. In contrast, individual interest reflects a student’s long-term stable characteristics; it is relatively less affected by the learning context (Hidi, 1990; Harackiewicz et al., 2000; Ainley, 2006).

Hidi and Renninger (2006) further proposed a four-phase model of learning interest, arguing that situational interest can be differentiated into triggered situational interest and maintained situational interest. Similarly, individual interest can be differentiated into emerging individual interest and well-developed individual interest. Learning interest may be developed in sequence from triggered situational interest to maintained situational interest; it also has the potential to transform into emerging individual interest and finally into a well-developed individual interest. Learning interest may disappear or decline before the formation of well-developed individual interest. Linnenbrink-Garcia et al. (2010) conducted a study on middle school and college students to examine the construct validity of learning interest in mathematics and introduction-to-psychology courses, finding that students’ learning interest was composed of triggered situational interest, maintained situational interest (feeling dominant), and maintained individual interest (focused on task value) regardless of school level and subject domain. Harackiewicz et al. (2008) implemented a 4-year longitudinal study on college students in introduction-to-psychology courses, finding that triggered situational interest positively predicted maintained situational interest 13 weeks later and, in turn, maintained situational interest positively predicted individual interest seven semesters later. In general, relatively few studies have examined these three components of learning interest above the middle-school level. Wu (2022a) conducted a study to investigate kindergartener learning interest in minority-language course learning by comparing one-factor (i.e., learning interest), two-factor (i.e., situational interest and individual interest), and three-factor models (i.e., triggered situational interest, maintained situational interest, and individual interest), finding that kindergarteners’ learning interest consisted of situational interest and individual interest. Accordingly, the development of refined learning interest may need more time and must be accompanied by physical and/or mental growth. For students at the kindergarten level, learning interest may be only differentiated into two components: situational interest and individual interest.

### Effects of typical achievement goals on learning outcomes

By adopting a typical achievement goal viewpoint (i.e., dichotomous, trichotomous, and 2 × 2 achievement goal frameworks), the mastery/mastery-approach goal has generally been linked to numerous positive learning outcomes, including learning interest (Harackiewicz and Elliot, 1993; Harackiewicz et al., 1997, 2000; Elliot et al., 2005), challenge appraisal, absorption during preparation, grade aspirations (McGregor and Elliot, 2002), intrinsic motivation, self-regulation learning, learning efficacy (Elliot and Church, 1997; Harackiewicz et al., 1997, 2002; Elliot, 2005), deep learning strategies such as elaboration (Elliot et al., 1999; Elliot and McGregor, 2001; Wolters, 2004), positive affect (Daumiller et al., 2021), academic adjustment (Wang et al., 2021), and mathematics performances (Barron and Harackiewicz, 2003; Linnenbrink, 2005; Ryan et al., 2005; Cury et al., 2006; Sideridis, 2006; Lau and Nie, 2008). The results clearly indicated that mastering learning tasks and devoting more cognitive resources and strategies may lead to better academic achievements. However, some studies have found null effects of the mastery/mastery-approach goal on mathematics performance (Wolters, 2004; Zusho et al., 2005; Young, 2007; Seaton et al., 2014), and it has even been found to undermine mathematics performance (Plante et al., 2013). In contrast, studies have consistently found that the mastery/mastery-approach goal is positively related to interest both in the short- and long term (Harackiewicz et al., 1997, 2000, 2002, 2008; Hulleman et al., 2008).

Compared with the mastery/mastery-approach goal, the effects of the performance-approach goal on learning outcomes are generally more complicated. The performance-approach goal has been positively linked to intrinsic motivation (Elliot and Harackiewicz, 1996; Elliot and Church, 1997) and more frequently leads to better academic achievement, higher text anxiety, and avoiding help-seeking behavior than the mastery/mastery-approach goal (Elliot and Church, 1997; Harackiewicz et al., 1997; Elliot, 1999; Elliot et al., 1999, 2011; Elliot and McGregor, 1999; Harackiewicz et al., 2000; Barron and Harackiewicz, 2003; Ryan et al., 2005; Sánchez-Rosas and Pérez, 2015; Elliot and Hulleman, 2017). In addition, the performance-approach goal has also been positively linked to surface learning strategies, such as rehearsal (Elliot et al., 1999; Escribe and Huet, 2005). However, some studies have found that it has detrimental effects on intrinsic motivation (Rawsthorne and Elliot, 1999) or null effects on learning interest and mathematics performances (Harackiewicz and Elliot, 1993; Harackiewicz et al., 1997, 2000; Linnenbrink, 2005; Ryan et al., 2005; Gutman, 2006; Sideridis, 2006; Young, 2007; Lau and Nie, 2008; Keys et al., 2012).

In terms of mastery- and performance-avoidance goals, studies have consistently found detrimental or null effects on positive learning outcomes; they have also been positively linked to the surface or negative learning processes. Specifically, the mastery-avoidance goal has been found to positively predict test anxiety (Elliot and McGregor, 2001) but negatively predict the adaptation of deep learning strategy, interest (composed of both situational and individual interest; Harackiewicz et al., 2000), and mathematics performance (Zusho et al., 2005; Young, 2007). The performance-avoidance goal has been positively linked to surface learning strategy, text anxiety, and self-handicapping strategy; it has also been negatively linked to learning interest, help-seeking behavior, academic adjustment, and mathematics performance (Middleton and Midgley, 1997; Ryan et al., 2005; Young, 2007; Hulleman et al., 2010; Huang, 2011; Sánchez-Rosas and Pérez, 2015; Elliot and Hulleman, 2017; Wang et al., 2021). Some studies have found null effects on mathematics performance (Wolters, 2004; Linnenbrink, 2005; Ryan et al., 2005; Cury et al., 2006; Sideridis, 2006; Lau and Nie, 2008; Seaton et al., 2014).

In general, the mastery- and performance-avoidance goals may lead to maladaptive learning outcomes (e.g., declined learning interest and performed worse or with no benefits on mathematics exams). Similarly, the positive effects of the mastery/mastery-approach goal and the null effects of the performance-approach goal on learning interest have been consistently identified by many studies. However, these effects on learning interest are not conclusive because the interest measurements used in these studies were either situational interest (e.g., Harackiewicz et al., 1997) or a mix of both interest components (i.e., situational and individual interest), which formed a single indicator (e.g., Harackiewicz et al., 2000). Finally, the effects of the mastery- and performance-approach goals on mathematics performances are inconclusive. More importantly, the above findings were obtained based on school-aged students and typical achievement goal framework, and the learning interests and mathematics performances of these students were closely connected to formal learning. Accordingly, the results cannot be generalized to kindergartener samples in informal education contexts and to the effects of the 3 × 2 achievement goal framework.

### Impact of 3 × 2 achievement goals on learning outcomes: empirical findings

The literature review suggests that the task-approach goal is positively linked to perceived anxiety control, learning efficacy, absorption in class, emotional recognition, deep learning strategy, intrinsic motivation, satisfaction, engagement, positive affect, perceived competence, empathy, emotional control, instrumental help-seeking behavior, mathematical-modeling competency, and incremental beliefs (Elliot et al., 2011; Gillet et al., 2015; Hoi, 2016; Méndez-Giménez et al., 2017; Wang et al., 2017; Hidayat et al., 2018; Méndez-Giménez et al., 2018; Danthony et al., 2020). In addition, the task-approach goal has not been linked to bodily symptoms of anxiety, exam anxiety, exam performance, energy in class, deep strategy, and surface strategy (Elliot et al., 2011; Gillet et al., 2015; Hoi, 2016; Danthony et al., 2020), and it has been negatively linked to self-focus anxiety, somatic tension anxiety, problem-solving skills, and entity beliefs (Wang et al., 2017; Danthony et al., 2020; Liu and Liu, 2020).

The self-approach goal has been positively linked to perceived anxiety control, energy in class, intrinsic motivation, satisfaction, engagement, perceived competence, empathy, emotional control, deep strategy, mathematical-modeling competency, and incremental beliefs (Elliot et al., 2011; Gillet et al., 2015; Hoi, 2016; Méndez-Giménez et al., 2017, 2018; Wang et al., 2017; Hidayat et al., 2018; Danthony et al., 2020). In addition, the self-approach goal does not relate to exam anxiety, exam performance, emotional recognition, exam anxiety, deep strategy, surface strategy, and instrumental help-seeking behavior (Elliot et al., 2011; Gillet et al., 2015; Hoi, 2016), and it has been negatively linked to self-focus anxiety, bodily-symptoms anxiety, somatic tension anxiety, problem-solving skills, and entity beliefs (Wang et al., 2017; Danthony et al., 2020; Liu and Liu, 2020). Studies have linked the other-approach goal to some positive learning outcomes, such as autonomy, learning efficacy, perceived competence, and academic achievement (Elliot et al., 2011; Mascret et al., 2015; Hoi, 2016; Méndez-Giménez et al., 2017), but it has also been negatively linked to some adaptive learning outcomes, such as autonomy and help-seeking behavior (Yang et al., 2016; Méndez-Giménez et al., 2017).

In terms of the three avoidance-based achievement goals (i.e., task-avoidance goal, self-avoidance goal, and other-avoidance goal), they have rarely been linked to positive learning outcomes (e.g., deep strategy, class absorption, individual interest, or executive help-seeking behavior), but they have been frequently negatively linked to several positive learning outcomes (e.g., exam performance, autonomy, and mathematical-modeling competency) or positively linked to negative learning processes, such as test anxiety (Gillet et al., 2015; Hoi, 2016; Yang et al., 2016; Hidayat et al., 2018; Danthony et al., 2020).

Overall, similar to the studies adopting typical achievement goal frameworks, the detrimental or null effects of these three avoidance-based achievement goals are relatively consistent. However, the effects of three approach-based goals on learning outcomes are inconclusive. More importantly, the above findings can only be applied to students above elementary school levels, and their effects on learning interest and mathematics performances have been under investigated. Consequently, to enrich the literature, studies are required based on kindergarten samples. In doing so, kindergarten teachers and policymakers will become aware of which achievement goals are the most adaptive for learning outcomes and thus should be cultivated.

## Current study

The effects of achievement goals on learning interests and mathematics performances in early years may have a profound impact on future learning consequences. For example, the effects of adaptive achievement goals (e.g., the mastery-approach goal) on learning interest in mathematics and mathematics performances in kindergarten may promote learning interest in mathematics and lead to better mathematics performances. In turn, these interests and mathematics performances may contribute to better academic achievement and positive learning motivation in later years (Jordan et al., 2010; Fisher et al., 2012). However, no studies have investigated the effects of achievement goals on learning interests and mathematics performances for kindergarteners based on the new 3 × 2 achievement goal framework. More studies are required for both researchers and kindergarten teachers to understand what achievement goals may be beneficial in terms of facilitating learning interest and mathematics performance in less structured kindergarten classrooms with informal learning contexts.

The main purpose of this study is as follows:

1. to investigate the effects of 3 × 2 achievement goals on learning interest and mathematics performances.

Because existing studies adopted either typical or the latest achievement goal frameworks, learners who were oriented to achieve task requirements may have been interested and immersed in learning tasks. They would also have been more likely to use deep learning strategies rather than surface learning strategies to resolve problems. Therefore, the following effects can be expected:

*Hypothesis 1a*. The task-approach goal has positive effects on both situational and individual interests and mathematics performance involving deep learning strategies (e.g., advanced arithmetic for kindergarteners). Hypothesis 1b. The task-approach goal has no significant effects on mathematics performance involving surface learning strategies (e.g., counting numbers in order).

The self-approach goal motivates students to perform better compared with past performances, whereas the focus of learning interest and mathematics performance is mainly on enjoying the teaching activities and learning tasks or correctly answering/solving mathematics problems. In addition, the positive and negative effects of the self-approach goal are yet to be identified on adaptive and maladaptive learning outcomes, respectively, but some studies have found null effects on exam performance. Therefore, the following effects can be expected:

*Hypothesis 2*. The self-approach goal has no significant effects on learning interests and mathematics performance.

For the other-approach goal, several studies have found its null effects on learning interest and mathematics performances (e.g., Harackiewicz et al., 1997, 2000; Gutman, 2006), but other studies have indicated positive effects on academic achievement (e.g., Barron and Harackiewicz, 2003; Sánchez-Rosas and Pérez, 2015). In addition, the other-approach goal has been positively linked to surface learning strategies (e.g., Escribe and Huet, 2005). Taken together, the following effects of the other-approach goal can be expected:

*Hypothesis 3a*. The other-approach goal has no significant effects on learning interests. Hypothesis 3b. The other-approach goal has positive effects on mathematics performance involving surface learning strategies, but it has no significant effects on mathematics performance involving deep learning strategies.

After integrating the findings related to avoidance-based goals, it is evident that they are usually positively related to maladaptive learning outcomes or negatively related to adaptive learning processes. This suggests that avoidance motivation may exert independent negative effects regardless of the referents (i.e., absolute, intrapersonal, or interpersonal) that learners use to define their competence. However, avoidance motivation may be increased with failure experiences. Moreover, the ratings for avoidance-based goals are lower than those for approach-based goals for students in elementary school and junior high school (Bong, 2009). Therefore, for kindergarteners, avoidance motivation may not have salient effects alone; rather, its effects on learning outcomes may be dependent on the goal foci themselves. Specifically, the positive effects of task foci may relieve the potentially detrimental effects of avoidance motivation on learning interests and mathematics performances regardless of the strength of avoidance motivation. For the self-foci goal, the null effects on learning interests and mathematics performances may be undermined by avoidance motivation, and its effects may be overturned. In addition, the other-avoidance goal has generally been negatively related to learning interest and exam performance. However, the effects of the self-avoidance goal and the other-avoidance goal are trivial when the strength of said goals is low. Consequently, the following effects can be expected for avoidance-based goals:

*Hypothesis 4*. The task-avoidance goal has positive effects on learning interests and mathematics performances involving deep learning strategies.

*Hypothesis 5*. The self-avoidance goal has slightly negative effects on both learning interests and mathematics performances when the strength of the self-avoidance goal is high, but the effects are trivial when the strength is low.

*Hypothesis 6*. The other-avoidance goal has slightly negative effects on both learning interests and mathematics performances when the strength of the other-avoidance goal is high, but the effects are trivial when the strength is low.

## Methodology

### Participants

A total of 180 (89 boys and 91 girls) kindergarteners aged 5 years old and 15 kindergarten teachers (selected from 15 kindergartens in Taiwan) consented to participate in the study. Participants were assured that all their responses would be kept confidential, and kindergarteners and their parents were informed that their treatment by teachers would not be influenced once they participated.

### Instruments

Three measurements were used in this study: the achievement goal scale, learning interest scale, and mathematics performance test.

#### Achievement goal scale

Wu (2022b) has developed a valid measurement for investigating kindergarteners’ achievement goals. Two versions have been designed for kindergarteners’ self-reporting and for kindergarten teachers to rate kindergarteners in their classroom, both of which have been demonstrated as valid tools for evaluating kindergarteners’ achievement goals. The teacher version was adopted because it is more time effective (by a factor of seven) than the self-reporting version. In doing so, any disturbance to practices due to this study was expected to be reduced.

The achievement goal scale consists of 18 items designed to measure six achievement goal dimensions based on the 3 × 2 achievement goal model. There are six subscales with three items in each. Pseudonyms were used for the items, which had no effect on kindergarten teachers’ ratings for boys and girls. The sample items to measure each achievement goal are as follows: (1) task-approach goal: John is absorbed in building a castle in the block area; (2) self-approach goal: John tells the teacher “I want to build a castle that is higher than I have made in the past;” (3) other-approach goal: John competes with Bob and says “I want to build a castle higher than yours;” (4) task-avoidance goal: John ran away from the block area because he could not build a castle well; (5) self-avoidance goal: John tells Bob “I do not want to stack up blocks lower than I have made in the past;” and (6) other-avoidance goal: John competes with Bob and says “I do not want to stack up blocks lower than yours” (Wu, 2022b). Teachers were required to evaluate the correspondence for item descriptions with their observations for each kindergartener, and they had to choose one option for each kindergartener on a 6-point Likert scale: 6 (totally in line with them), 5 (largely in line with them), 4 (partly in line with them), 3 (small discrepancies between them), 2 (large discrepancies between them), and 1 (completely dissimilar to them).

#### Learning interest scale

The present article adopted and revised the learning interest scale for specific language-learning courses for kindergarteners developed and validated by Wu (2022a). The learning interest scale consists of two subscales: situational interest and individual interest. Both subscales consist of three items, and the same design as the achievement goal scale was used. The sample item for situational interest was “Bob feels that teaching activities are interesting.” The sample item for individual interest was “Bob is looking forward to the teaching activities coming.” Teachers were required to choose one option for each kindergartener on a 6-point Likert scale: 6 (totally in line with them), 5 (largely in line with them), 4 (partly in line with them), 3 (small discrepancies between them), 2 (large discrepancies between them), and 1 (completely dissimilar to them).

#### Mathematics performance test

The mathematics performance test was developed by referring to Ginsburg and Baroody (2003). This test consisted of of three subtests, including the numbering and counting subtest, the basic arithmetic subtest, and the advanced arithmetic subtest. The numbering and counting subtest included seven items; the sample item was “Verbally count a certain number of apples.” The basic arithmetic subtest included six items; the sample item was “Mother buys seven apples, but two of them were eaten by Bob. How many apples does mother have now?” The advanced arithmetic subtest included four items; the sample item was “Mother brings 16 apples home, but some apples were eaten by Bob. Finally, there were nine apples left. How many apples were eaten by Bob?” The internal consistency reliabilities of all items in the mathematics performance test are >0.90.

The kindergarteners were allowed to use objects to solve mathematical problems. A score of 1 was assigned to correct answers, representing kindergarteners had mastered the concept in the test, and a score of 0 was assigned for incorrect answers. The overall internal consistency reliability of the mathematics performance test is 0.88. The internal consistency reliability of the numbering and counting subtest is 0.79. The internal consistency reliability of the basic arithmetic subtest is 0.86. The internal consistency reliability of the advanced arithmetic subtest is 0.71.

### Analysis

Confirmatory factor analyses were conducted to re-validate/validate the scales used in this study by recruiting different samples to reassure their effectiveness in terms of kindergartener achievement goals and learning interests. The following indices were used to validate these two scales: the chi-square statistic (χ^2^), the comparative fit index (CFI), the Tucker–Lewis index (TLI), and the root mean square error of approximation (RMSEA). The lower the *χ*^2^-value, the better the model fit. Ideally, a non-significant *χ*^2^-value represents that the null hypotheses of measurement models for achievement goal and learning interest (fitted to the observations) cannot be rejected. However, *χ*^2^ statistics are affected by sample size: the larger the sample size, the easier it is to reject the hypotheses of model fit (Wang and Wang, 2012). The remaining indices were taken as the main criteria to evaluate the goodness of fit of the measurement models. For these, a good fit between the hypothesized model and observations is indicated when CFI and TLI are approximal to 0.95 and RMSEA is approximal to 0.06 (Hu and Bentler, 1999). Convergent validity is verified if the composite reliability (CR) and average variance extracted (AVE) are greater than and equal to 0.60 and 0.50, respectively (Hair et al., 2009). The discriminant validity is preliminarily demonstrated if the bootstrapped 95% confidence intervals (95% CI) of the inter-correlation coefficients among all factors are <1 (Torkzadeh et al., 2003).

**Figure 1:**
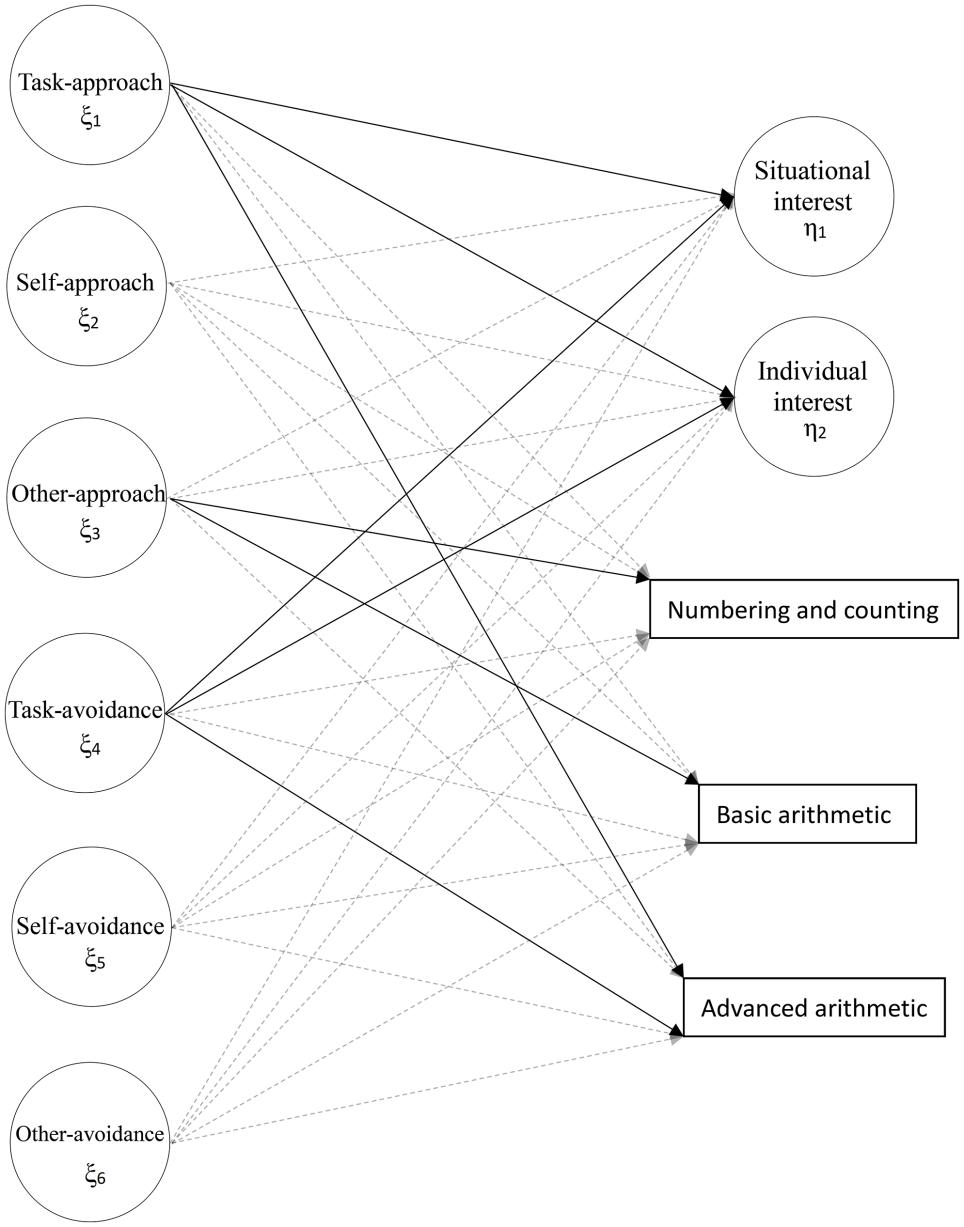
Hypothesized effects of achievement goals on learning interest and mathematics performances. The circles represent latent variables, while the rectangles represent indicators. The observations for each latent variables and residual variances are not shown for simplicity.

After validating the measurement models, the structural model, which includes the relationships between the exogenous variables (i.e., the six achievement goals) and endogenous variables (i.e., two learning interests and three mathematics performances), was analyzed based on the above criteria and cutoff values to evaluate its goodness of fit. Figure 1 shows the hypothesized effects of achievement goals on learning interest and mathematics performances, from which it is evident that the six achievement goals point toward the two learning interest indicators and the three mathematics-performance indicators.

## Results

### The goodness of fit of measurement models

The results showed that the original six-factor achievement goal model (composed of 18 items in total with each factor measured via three items) does not fit the data: χ^2^(120, *N* = 180) = 394.15, *p* < 0.05, CFI = 0.85, TLI = 0.81, RMSEA = 0.113 (90% CI ranged from 0.100 to 0.125). However, after deleting three items with lower factor loadings (i.e., the resulting percentages of explained variances by the item in a latent variable are less than 50%—that is, the 6th, 10th, and 14th items developed for measuring the self-approach goal, the task-avoidance goal, and the self-avoidance goal, respectively), the revised six-factor achievement goal model fit the data well: χ^2^(75, *N* = 180) = 145.62, *p* < 0.05, CFI = 0.95, TLI = 0.93, and RMSEA = 0.072 (90% CI ranged from 0.055 to 0.090). The three items removed included the self-approach goal, the task-avoidance goal, and the self-avoidance goal. The CRs for the six achievement goal factors ranged from 0.75 to 0.95, and were thus all greater than the cutoff value of 0.60. The AVEs for the six achievement goal factors ranged from 0.59 to 0.88 and were thus all greater than the cutoff value of 0.50. The bootstrapped 95% CI of the inter-correlation coefficients among six factors ranged from −0.26 to 0.92. The bootstrapped 95% CIs of inter-correlation coefficients among six factors did not include 1.

The two-factor learning interest model fitted to the data: χ^2^(8, *N* = 180) = 6.80, *p* > 0.05, CFI = 1.00, TLI = 1.00, and RMSEA = 0.000 (90% CI ranged from 0.000 to 0.078). The CRs for the two factors are, respectively, 0.94 and 0.95, both of which are greater than the cutoff value of 0.60. The AVEs for the two factors are, respectively, 0.84 and 0.86, both of which are greater than the cutoff value of 0.50. Bootstrapped 95% CI of the inter-correlation coefficients among the six factors ranged from 0.85 to 0.97. The bootstrapped 95% CI of inter-correlation coefficient among two factors did not include 1.

Taken together, the results suggest that the achievement goal scale and learning interest scale for kindergarteners have good validities and internal structural qualities, and thus they are effective when used to investigate kindergarteners’ achievement goals and learning interests.

### Effects of achievement goals on learning interests and mathematics performances

**Figure 2:**
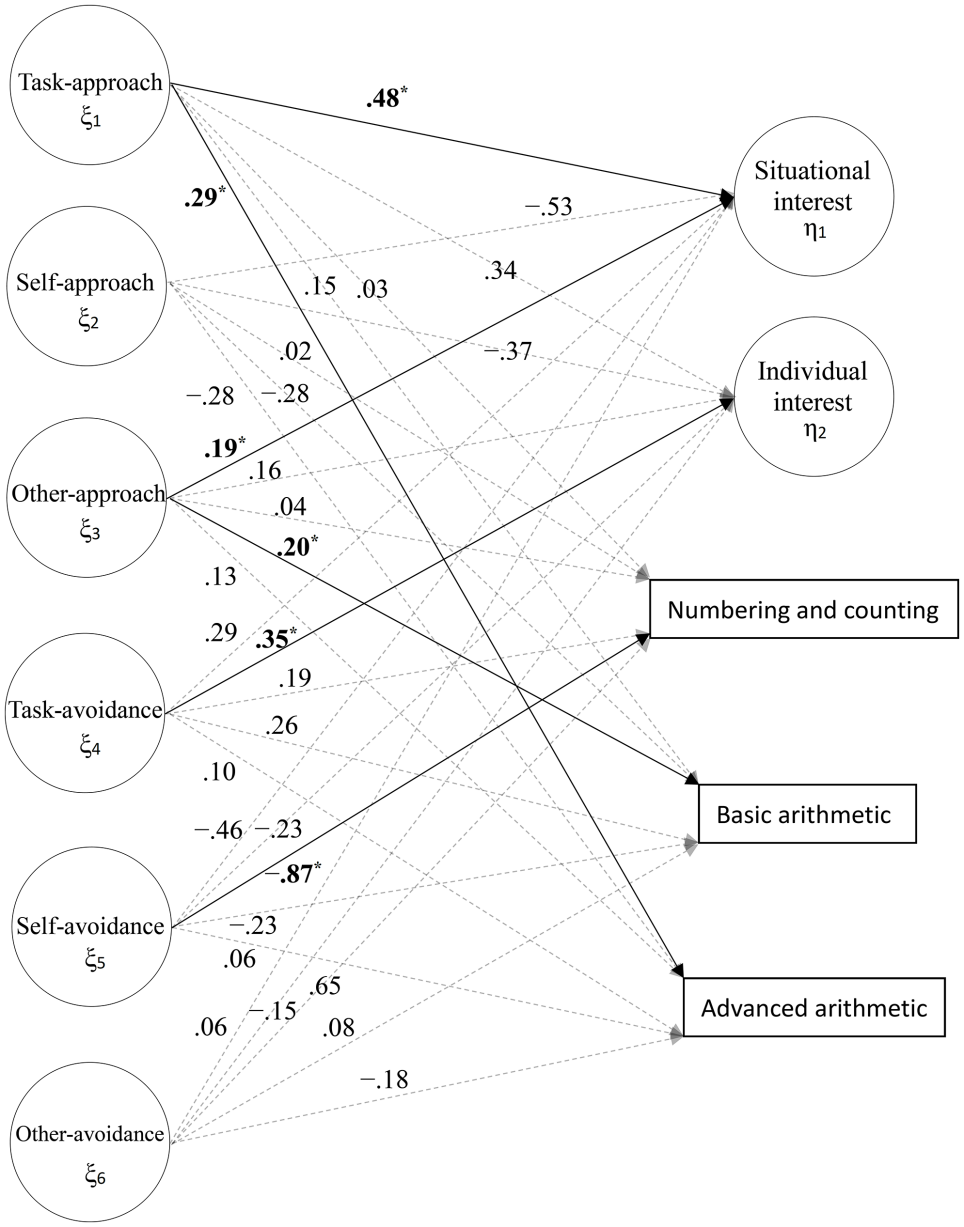
Empirical effects of achievement goals on learning interest and mathematics performances. The solid lines and arrows with bold signs, numbers, and asterisks indicate significant effects, whereas the semi-translucent dotted lines and arrows with normal signs and numbers and without asterisks indicate non-significant effects. The coefficients are completely standardized. ^*^p < 0.05.

The structural model was analyzed based on good-fit measurement models. Table 1 presents the effects of achievement goals on learning interest and mathematics performances, which are also visualized in Figure 2. As can be seen in Table 1 and Figure 2, the task-approach goal positively predicted situational interest and advanced arithmetic performance (*β*_11_ = 0.48 and *β*_15_ = 0.29, *p* < 0.05), but it had no effect on individual interest, numbering and counting performance, and basic arithmetic performance (*β*_12_ = 0.34, *β*_13_ = 0.03, and *β*_14_ = 0.15, *p* > 0.05). The self-approach goal had no effect on situational interest, individual interest, numbering and counting performance, basic arithmetic performance, and advanced arithmetic performance (*β*_21_ = −0.53, *β*_22_ = −0.37, *β*_23_ = 0.02, *β*_24_ = −0.28, and *β*_25_ = −0.28, *p* > 0.05). The other-approach goal positively predicted situational interest and basic arithmetic performance (*β*_31_ = 0.19 and *β*_34_ = 0.20, *p* < 0.05), but it had no effect on individual interest, numbering and counting performance, and advanced arithmetic performance (*β*_32_ = 0.16, *β*_33_ = 0.04, and *β*_35_ = 0.13, *p* > 0.05). The task-avoidance goal positively predicted individual interest (*β*_32_ = 0.35, *p* < 0.05), but it had no effect on situational interest, numbering and counting performance, basic arithmetic performance, and advanced arithmetic performance (*β*_41_ = 0.29, *β*_43_ = 0.19, *β*_44_ = 0.26, and *β*_45_ = 0.10, *p* > 0.05). The self-avoidance goal negatively predicted numbering and counting performance (*β*_53_ = −0.87, *p* < 0.05), but it had no effect on situational interest, individual interest, basic arithmetic performance, and advanced arithmetic performance (*β*_51_ = −0.46, *β*_52_ = −0.23, *β*_54_ = −0.23, and *β*_55_ = 0.06, *p* > 0.05). The other-avoidance goal had no effect on situational interest, individual interest, numbering and counting performance, basic arithmetic performance, and advanced arithmetic performance (*β*_61_ = 0.06, *β*_62_ = −0.15, *β*_63_ = 0.65, *β*_64_ = 0.08, and *β*_65_ = −0.18, *p* > 0.05).

**Table 1:** The effects of achievement goals on learning interest and mathematics performances.

Variables	Situational interest (1)	Individual interest (2)	Numbering and counting (3)	Basic arithmetic (4)	Advanced arithmetic (5)
Task-approach	0.48^*^	0.34	0.03	0.15	0.29^*^
Self-approach	−0.53	−0.37	0.02	−0.28	−0.28
Other-approach	0.19^*^	0.16	0.04	0.20^*^	0.13
Task-avoidance	0.29	0.35^*^	0.19	0.26	0.10
Self-avoidance	−0.46	−0.23	−0.87^*^	−0.23	0.06
Other-avoidance	0.06	−0.15	0.65	0.08	−0.18

The coefficients are completely standardized. ^*^p < 0.05.

## Discussion

The effects of achievement goals on learning interests and mathematics performances can only be clarified based on effective measurements. At first, this study re-examined the construct validity, convergent validity, and discriminant validity of achievement goal and learning interest for kindergarteners by adopting the newest and most recently validated measurements (Wu, 2022a,b). The validities of these two constructs were demonstrated again in this study with respect to a different kindergarten sample. The results indicated that both the six-factor achievement goals and two-factor learning interests were suitable for understanding kindergarteners’ achievement goals and learning interests, which re-confirmed our former findings.

The task-approach goal had positive effects on situational interest and advanced arithmetic performance but no effects on individual interest, numbering and counting, and basic arithmetic performances. The expected effects of the task-approach goal on learning interests and mathematics performances are largely supported by the above findings, except for the effect on individual interest. It was noted that individual interest is elicited by inner processes (i.e., the affect and value of the learning task), whereas situational interest is triggered by environmental stimuli (Hidi, 1990). The task-approach goal orients students to master the learning task, and, in this context, it is reasonable to direct student attention to the learning task. However, individual interest needs to be developed based on the long-term positive affect and recognition of the values that the learning task offers. Therefore, the task-approach goal only contributed to triggering situational interest, which is consistent with some former studies (e.g., Harackiewicz et al., 1997) but not others (e.g., Harackiewicz and Elliot, 1993). In addition, deep learning strategies can help learners to transfer the knowledge obtained to solve different problems (Floyd et al., 2009); it is required to solve problems related to advanced arithmetic. The task-approach goal was found to promote the use of deep learning strategies. Therefore, it was reasonable to have a positive effect solely on advanced arithmetic performance, which is consistent with several former studies that did not differentiate mathematics performance into different abilities involving deep or surface learning strategies (e.g., Barron and Harackiewicz, 2003; Linnenbrink, 2005; Ryan et al., 2005; Cury et al., 2006; Sideridis, 2006; Lau and Nie, 2008). In other words, only the task-approach goal contributes to advanced arithmetic performance, but, after considering the effects of other achievement goals related to surface learning strategies, it is evident that it has no additional effects on mathematics performances using said learning strategies. The latter findings are similar to studies that found no effects of the task-approach goal on mathematics performance (Wolters, 2004; Zusho et al., 2005; Young, 2007; Seaton et al., 2014).

The self-approach goal was found to have no effects on both learning interests and mathematics performances, which is consistent with the expectations and arguments of the present study. These results are partially consistent with former studies that found no effects of the self-approach goal on exam performance (Elliot et al., 2011; Gillet et al., 2015; Hoi, 2016), but they contradict others studies claiming it has a positive effect on mathematics ability (Hidayat et al., 2018). This may be due to different foci among the self-approach goal, two learning interests, and mathematics performances. Specifically, the focus of the self-approach goal is on the intrapersonal comparison, the focus of both situational interest and mathematics performances is on environmental stimuli, and the focus of individual interest is on the inner affect and values of the learning task. In addition, it is important to note that the self-approach goal had four non-significant negative effects on situational interest, individual interest, basic arithmetic performance, and advanced arithmetic performance. Further studies are required to examine whether the effects on these learning outcomes are detrimental in the long term.

It was expected that the other-approach goal would have no effects on learning interests, but the results indicated a positive effect and a null effect on situational interest and individual interest, respectively, which may be due to the fact that a social-comparison component existed in the learning environment, and this could have elicited situational interest in kindergarteners, whereby they became interested when competing with others in teaching and learning activities. In essence, the other-approach goal had no effect on individual interest because it was not used as a component to define this goal. Moreover, expectations regarding the positive effects of the other-approach goal on mathematics performances involving surface learning strategies were partially supported. This may be due to the ceiling effect of numbering and counting performance, which means that this goal has limited effects on such performance. However, there was no such ceiling effect on basic arithmetic performance, and the other-approach goal exerted a positive effect on this performance type. These findings partially correspond with expectations, indicating that the other-approach goal had no effect on individual interest and had positive effects on situational interest and basic arithmetic performance. This is consistent with the findings of several prior studies (Harackiewicz and Elliot, 1993; Harackiewicz et al., 1997, 2000; Linnenbrink, 2005; Ryan et al., 2005; Gutman, 2006; Sideridis, 2006; Young, 2007; Lau and Nie, 2008; Keys et al., 2012). However, the other-approach goal only had a positive effect on situational interest and no effect on individual interest, and thus further studies are required.

The task-avoidance goal had a positive effect on individual interest and no effect on the remaining learning outcomes. The expectations related to this goal were also partially supported. However, the non-significant effects of this goal on situational interest and mathematics performances and the positive effects on individual interest are inconsistent with former studies that suggest it has negative effects on learning interest and mathematics performances (Harackiewicz et al., 2000; Zusho et al., 2005; Young, 2007). In contrast, the null effects of this goal on mathematic performances are consistent with some studies (Elliot et al., 2011; Diseth, 2015). In addition to the significant positive effect on individual interest, the task-avoidance goal had an additional four positive but non-significant effects, which may suggest that the task-focus goal protects learning interest and mathematics performances from the potentially detrimental effects of avoidance motivation.

The self-avoidance goal had a negative effect on numbering and counting performances but no effect on the remaining learning outcomes. Thus, our expectations of this goal were not supported, except for the negative effect. From the mean values of the items measuring the self-avoidance goal (mean values close to 3), it is evident that they have middle strength. Theoretically, avoidance motivation may exert negative effects on these outcomes, but only numbering and counting performances were significantly affected. The effects of three (out of the remaining four outcomes) were non-significant and present negative tendencies. The null effects of the self-avoidance goal on basic arithmetic performance and advanced arithmetic performance are consistent with several former studies (Elliot et al., 2011; Diseth, 2015; Hoi, 2016; Didin and Kasapoglu, 2021). However, its null effects on learning interest are an original contribution to the achievement goal literature.

The other-avoidance goal had no effect on both learning interest and mathematics performance. This is consistent with our expectations because the mean values of the items measuring this goal are low and its effects on mathematics performance are similar to former studies (Wolters, 2004; Linnenbrink, 2005; Ryan et al., 2005; Cury et al., 2006; Sideridis, 2006; Lau and Nie, 2008; Seaton et al., 2014). However, at the same time, this finding contradicts several former studies, which found negative effects on learning interest and mathematics performance (Middleton and Midgley, 1997; Ryan et al., 2005; Young, 2007; Hulleman et al., 2010; Huang, 2011; Sánchez-Rosas and Pérez, 2015; Elliot and Hulleman, 2017).

## Implications

The task-approach goal, performance-approach goal, and task-avoidance goal had positive effects on situational interest, individual interest, basic arithmetic performance, and advanced arithmetic performance. They also had some positive but non-significant effects (e.g., the effects of the task-approach goal and the other-approach goal on individual interest). However, more studies are needed to re-examine the above findings. In particular, future research should examine whether the significant effects of these achievement goals are short- or long term and whether the non-significant positive effects will become significant as time passes.

This study also found that the task-approach goal had a positive effect on mathematics performances based on deep learning strategies but no effect on mathematics performances based on surface learning strategies (e.g., numbering and counting). These results may be unique, and future studies should subdivide mathematics performance to clarify the effects (and thus enhance understanding) of the task-approach goal on different instances of mathematics performances.

The self-approach goal, self-avoidance goal, and other-avoidance goal had non-significant but negative effects on learning interests and mathematics performances. This may suggest that there are no short-term effects on these learning outcomes, but it is unclear whether these effects might be detrimental in the long term. Therefore, researchers should conduct longitudinal studies to clarify whether said goals have long-term negative effects on learning interests and mathematics performances.

In general, the task-based goals and the other-approach goal had significant benefits on some aspects of learning interests and mathematics performances. For example, the task-approach goal is beneficial for advanced arithmetic performance, whereas the other-approach goal is beneficial for basic arithmetic performance. Moreover, according to the multiple goals viewpoint (i.e., the mastery goal and the performance goal combination; Pintrich, 2000), these achievement-goal patterns may also reinforce the original significant benefits (or add to non-significant effects) and contribute to the formation of new positive and significant effects on these learning outcomes. For example, the non-significant effects of the task-approach goal and the other-approach goal on individual interest may be, respectively, combined to reinforce the significant positive effect of the task-avoidance goal. Similarly, the combination of the two non-significant effects of the task-approach goal and the other-approach goal on individual interest may contribute to the formation of a new significant positive effect on individual interest. Consequently, future research should investigate this phenomenon further by adopting the multiple goals viewpoint and corresponding statistics (e.g., mixture analysis) to clarify the combined effects of achievement goals on learning interests and mathematics performances.

For practitioners, it may be beneficial to cultivate these achievement goal patterns (e.g., by combining the task-approach goal and the other-approach goal) by addressing task mastery and incorporating appropriate competitions in kindergarten classes. It has been demonstrated that teachers can effectively promote the task-approach goal through the TARGET framework (comprising the six dimensions: Task, Authority, Recognition, Grouping, Evaluation, and Time) to arrange the teaching and learning activities in the classroom (Lüftenegger et al., 2013). According to TARGET, teachers are encouraged to assign learning tasks that focus on mastering learning materials or content, appropriate task challenges, and active engagement (Task), and allow students to decide what they are interested in learning and to set their own pace to complete the work (Authority and Time). Furthermore, teachers should encourage kindergarteners to work in groups (Grouping) and should evaluate their progress in the work process and give proactive feedback to recognize their efforts (Recognition and Evaluation). For example, kindergarten teachers should allow kindergarteners to determine what to build using a selection of materials and encourage them to work in groups and in different ways according to their own pace and monitor their working progression and approve their work or give instructions or suggestions when they encounter difficulties completing the work. In addition, teachers can invite kindergarteners in each group to share their work and working experiences and to approve and highlight the advantages of each group to other groups to promote healthy competition and to provide opportunities for kindergarteners to learn from other models. Finally, practitioners should be aware of the possible negative effects of self-based goals and other-avoidance goals on learning interests and mathematics performances and, if necessary, alter the goals accordingly by adopting the above class arrangements.

## Summary

This study re-validated achievement goal and learning interest measurements and their corresponding theoretical frameworks based on a kindergarten sample and sequential cohort. The results suggested that the six-factor achievement goals and two-factor learning interests are the best viewpoints for understanding kindergartner achievement motivation and learning interest in practice, respectively. Moreover, the discriminant utilities of the task- and self-based goals recently proposed by researchers were re-confirmed by incorporating outcomes that have not yet been sufficiently investigated (i.e., learning interests), and differentiating mathematics performance types into those associated with deep and surface learning strategies. It was generally found that task-based goals have significant and often positive effects on learning interests and mathematics performances, especially for mathematics problems involving deep learning strategies. In contrast, the self-based goals had negative effects on these learning outcomes. The effects of the other-based goals were more complex. Specifically, the other-approach goal generally had positive but weak effects on learning outcomes, whereas the other-avoidance goal had non-significant positive effects on numbering and counting performance and two non-significant negative effects on individual interest and advanced arithmetic performance. The results suggested that self-based goals are beneficial for mathematics performances involving surface learning strategies. Finally, the effects of self-based goals and other-based goals on learning outcomes were dependent on avoidance motivation, and such goals based on avoidance motivation may sharpen their detrimental effects on learning outcomes.

## Limitations

In this study, the effectiveness and consistency of measurement results between kindergarteners’ self-reporting and teachers’ ratings of achievement goal were only demonstrated in the kindergarten sample (Wu, 2022a,b). The present study only demonstrated the effectiveness of teachers’ ratings of learning interest and identified two types of learning interests in kindergarteners. Therefore, the first limitation of this study centers around consistencies of measurement and dimensionality (i.e., measurement invariance) of learning interest in kindergarteners between results derived from teachers’ ratings and those from kindergarteners’ self-reporting. Consequently, results with regard to the effects of achievement goals on learning interest in kindergarteners should be interpreted and used with caution. Future studies are encouraged to develop a learning interest scale that enables kindergarteners to report their learning interest in certain learning activities by themselves (e.g., adopt a pictorial format to design items and read these item descriptions for kindergarteners to choose one option from a 4-point Likert scale), and to compare the results with this study.

Furthermore, this study serves as a preliminary study to clarify the effects of kindergarteners’ achievement goals on learning interest and mathematics performance in an Eastern country (i.e., Taiwan), which may limit the generalizability of the findings to kindergarten samples in other counties or cultures. Finally, some non-significant (though almost significant) effects of achievement goals were found in this study based on its small sample (i.e., 180 kindergarteners). These findings may not imply that these achievement goals have no effects on learning interests and/or mathematics performances; however, on the contrary, they may imply that these effects are difficult to detect in a relatively small sample. Significant effects for these achievement goals may be identified if future studies recruit a more representative and larger sample.

## Data availability statement

The original contributions presented in the study are included in the article/supplementary material, further inquiries can be directed to the corresponding author.

## Ethics statement

The studies involving human participants were reviewed and approved by the National Cheng Kung Human Subjects Institutional Review Board. Written informed consent to participate in this study was provided by the participants’ legal guardian/next of kin.

## Author contributions

CW agreed to be accountable for all aspects of the work in ensuring that questions related to the accuracy and integrity of any part of the work are appropriately investigated and resolved.

## Funding

This study was funded by the National Science and Technology Council (grant no. 111-2410-H-153-025-).

## Conflict of interest

The author declares that the research was conducted in the absence of any commercial or financial relationships that could be construed as a potential conflict of interest.

## Publisher’s note

All claims expressed in this article are solely those of the authors and do not necessarily represent those of their affiliated organizations, or those of the publisher, the editors and the reviewers. Any product that may be evaluated in this article, or claim that may be made by its manufacturer, is not guaranteed or endorsed by the publisher.
